# Antioxidant Active Phytochemicals in *Ternstroemia lineata* Explained by Aquaporin Mechanisms

**DOI:** 10.3390/plants13162223

**Published:** 2024-08-10

**Authors:** Nahim Salgado-Medrano, César Millán-Pacheco, Verónica Rodríguez-López, Lucía Corona-Sánchez, François Mesnard, Roland Molinié, Eleazar León-Álvarez, María Luisa Villarreal, Alexandre Toshirrico Cardoso-Taketa

**Affiliations:** 1 Centro de Investigación en Biotecnología, Universidad Autónoma del Estado de Morelos, Cuernavaca 62210, Mexico; nahim@uaem.mx (N.S.-M.); alvarez-leon2@hotmail.com (E.L.-Á.); 2 Facultad de Ciencias Biológicas, Universidad Autónoma del Estado de Morelos, Cuernavaca 62210, Mexico; 3 Facultad de Farmacia, Universidad Autónoma del Estado de Morelos, Cuernavaca 62210, Mexico; cmp@uaem.mx (C.M.-P.); veronica_rodriguez@uaem.mx (V.R.-L.); csl_ff@uaem.mx (L.C.-S.); 4 Unité Mixte de Recherche Transfrontalière (UMRT), Institut National de Recherche pour l’Agriculture, l’Alimentation et l’Environnement (INRAE), BioEcoAgro, Laboratoire BIOPI, University of Picardie Jules Verne, 80000 Amiens, France; francois.mesnard@u-picardie.fr (F.M.); roland.molinie@u-picardie.fr (R.M.)

**Keywords:** *Ternstroemia lineata* DC., Pentaphylacaceae, antioxidant activity, ABTS^•+^, H_2_O_2_–*Saccharomyces cerevisiae*, aquaporin, AQP3 model, AQP7, in silico

## Abstract

The antioxidant action of terngymnoside C (**1**) and hydroxytyrosol-1-glucoside (**2**), isolated for the first time from the flower buds of *Ternstroemia lineata*, as well as katsumadin (**3**), obtained from the seedless fruits, was evaluated using ABTS^•+^ and H_2_O_2_–*Saccharomyces cerevisiae* models. In silico docking analysis of **1**, **2**, and **3** determined their affinity forces to the aquaporin monomers of the modeled *S. cerevisiae* protein 3 (AQP3) and human protein 7 (AQP7) channels that regulate the H_2_O_2_ cell transport. The ABTS^•+^ antiradical capacity of these compounds showed IC_50_ values of 22.00 μM (**1**), 47.64 μM (**2**), and 73.93 μM (**3**). The *S. cerevisiae* antioxidant assay showed that at 25 µM (**1**) and 50 µM (**2** and **3**), the cells were protected from H_2_O_2_-oxidative stress. These compounds, together with quercetin and vitamin C, were explored through the modeled *S. cerevisiae* AQP3 and human AQP7 by molecular docking analysis. To explain these results, an antioxidant mechanism for the isolated compounds was proposed through blocking H_2_O_2_ passage mediated by aquaporin transport. On the other hand, **1**, **2,** and **3** were not cytotoxic in a panel of three cancer cell lines.

## 1. Introduction

In Mexican traditional medicine, dried flowers and fruit infusion of *Ternstroemia lineata* DC. (Pentaphylacaceae), one of the most important medicinal plants commercialized in Mexico, is widely used for nervous disorders, insomnia, cough, and rheumatic pain [1,2,3]. Nevertheless, its use in diseases such as rheumatoid arthritis, which is closely linked to oxidative stress, has aroused great interest in the knowledge of its active antioxidant principles and the involved mechanisms of action [4,5]. This species was reported early as *T. pringlei*, but a revision by experts concluded that the material collected at Huitzilac Municipality in Morelos State, Mexico, belongs to *T. lineata*. In addition, the genus *Ternstroemia* was reclassified within the family Pentaphylacaceae [6].

Glycosylated phenylethanoids, named ternstrosides A–F, were isolated from *Ternstroemia japonica* [7] and showed antioxidant action by inhibiting the hydroxyl radical, total ROS generation in the rat kidney post-microsomal fraction, and the peroxynitrile. Other species of the genus, e.g., *Ternstroemia sylvatica*, was investigated as an antioxidant, possessing an active methanolic extract in the 1,1-diphenyl-2-picrylhydrazyl (α,α-diphenyl-β-picrylhydrazyl) (DPPH) radical-scavenging and ferric reducing/antioxidant power (FRAP) assays [8]. The *Ternstroemia* genus is an interesting source of phenylethanoids that have demonstrated a wide range of pharmacological activities. For example, terngymnosides A–D from *Ternstroemia gymnanthera* showed a significant analgesic effect compared to aspirin in a mouse model [9].

The antioxidant effect of natural products can be mediated by water channel proteins called aquaporins (AQP), which are a family of small and integral membrane proteins occurring in animals and plants, responsible for water transport across cell membranes [10]. Wragg [11] demonstrated that H_2_O nanoconfinement within AQP3 has a bidirectional flux through single-file chains and revealed that H_2_O_2_ is able to mimic H_2_O during AQP3 peroxiporin permeation.

The main objective of the present work was to study the antioxidant capacity and the possible mechanism of action of two phenyletanoids, terngymnoside C (**1**) and hydroxytyrosol-1-glucoside (**2**), as well as the phenypropanoid katsumadin (**3**), isolated from *T. lineata*. For this purpose, the ABTS^•+^ scavenger, the in vivo model of *S. cerevisiae* subjected to oxidative stress with H_2_O_2_, and in silico analysis of the molecular docking with aquaporins from *S. cerevisiae* AQP3 and the human AQP7 were used. The compound quercetin, widely documented as a regulator of H_2_O_2_ transport by aquaporins, was used as a control for the in vivo and in silico experiments [12] as well as vitamin C, which, as far as we know, has not been related to the regulation of H_2_O_2_ through aquaporins but presents extensive documentation about its antioxidant properties.

We proposed one of the possible mechanisms of antioxidant action of compounds **1**, **2**, and **3** that may occur at the cell membrane level. This mechanism was considered based on in silico modeling, which involves delaying aquaporin-mediated H_2_O_2_ transport and maintaining cell viability. We observed a relationship between the percentages of cell viability recorded in the in vivo antioxidant assay and the affinity forces exerted by the isolated compounds, quercetin, and vitamin C on the pores of the protein monomers of the *S. cerevisiae* AQP3 and human AQP7 channels regulating H_2_O_2_ cell internalization. The present study evaluates the in vivo antioxidant capacity of compounds **1**, **2**, and **3** for the first time. Additionally, we explored the cytotoxic capacity of the isolated compounds using three cancer cells and a normal fibroblast cell line.

## 2. Results

### 2.1. Isolation and Identification of Compounds **1**, **2**, and **3**

**Figure 1:**
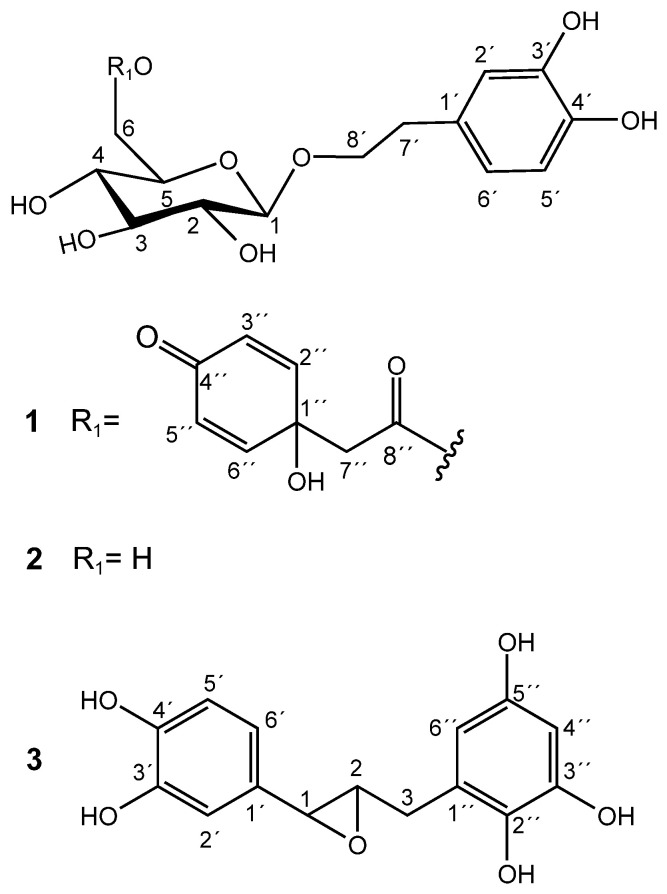
Chemical structures of **1**, **2**, and **3**.

Methanolic extracts of flower buds were subjected to chromatographic processes to afford pure compounds **1** and **2**. Purification of **1** resulted in a pale-yellow oil with a negative optical rotation value [α]^22^_D_: −17.2 (c 0.2, MeOH). The molecular formula C_22_O_11_H_26_ was estimated by the molecular ion [M+H]^+^ 467.1547 (*m*/*z* calculated of 467.1553) and by the adduct [M+Na]^+^ 489.1372 (*m*/*z* calculated of 489.1373), calculated through positive UHPLC-ESI-QTOF-MS analysis (Figure S1). The carbon number was corroborated by ^13^C NMR and DEPT 135 spectra. ^1^H NMR of **1** revealed the presence of a 3,4-dihydroxyphenethyl alcohol group, which was corroborated by the characteristic couplings of an ABX-type aromatic spin system for H-2′ (δ 6.68, d, 2.1 Hz), H-5′ (δ 6.66, d, 8.0 Hz), and H-6′ (δ 6.54, dd, 8.0, 2.1 Hz), as shown in Table S1 and Figure S2. Downfield chemical shifts for the backbone aromatic carbons at δ146.31 (C-3′) and δ144.80 (C-4′) also indicated a dihydroxy substitution at the phenethyl group. A C-7′ methylene group (δ 36.74; H-7′a/b, δ 2.77) was located in the β position of the ethyl group chain, which is also composed of an α-methylene group at C-8′ (δ 72.25; H-8a′, δ 3.68; and H-8b′, δ 4.02) bonded to an oxygen atom. A β-D-glucose unit was unambiguously assigned through characteristic chemical shifts of its anomeric proton at δ 4.29 (1H, d, 8 Hz) and carbon at δ 104.54. Sugar stereochemistry was established through the analysis of ^3^*J*_HH_ coupling constants (Table S1). The glycosidic bond between the sugar and the aglycone was determined through the long-distance correlation of ^2–3^*J*_HC_ (HMBC) between the anomeric H-1 glucose and the α-methylene carbon at C-8′, evidencing the glycosidic ether bond (Figure S3). Spectroscopic and spectrometric analysis and data comparison to the literature allowed the identification of **1** as terngymnoside C, a glycosylated quinoidal phenylethane-type molecule that was previously isolated from *Ternstroemia gymnanthera* [9], as shown in Figure 1.

Compound **2** was isolated as a pale-yellow oil and had a negative optical rotation value of [α]_22_^D^: −22.8 (c 0.3, MeOH). Its molecular formula C_14_O_8_H_20_ was estimated from the molecular ion [M−H]^−^ of *m*/*z* 315.10913 (*m*/*z* calculated of 315.10799) and from the adduct [M+Na]^+^ of *m*/*z* 339.34766 (*m*/*z* calculated of 339.10559) that were obtained from a high-resolution ESI-QTOF-MS spectrometer (Figure S4). The carbon number coincided with the information obtained by the decoupled ^13^C NMR analysis. The main ^1^H NMR signals observed for compound **2** were explained by the presence of the same characteristic ABX-type aromatic spin system and a β-D-glucose unit found in compound **1**, as shown in Table S1 and Figure S5. In fact, the only difference between **1** and **2** is the presence of the esterified quinol group in **1**. Thus, the NMR data of **2** agreed with that of the phenylethanoid 2-(3,4-dihydroxy)-phenylethyl-O-β-D-glucopyranoside, also known as hydroxytyrosol-1-glucoside and as hydroxysalidroside. Salidroside derivatives were isolated for the first time from the bark of *Salix triandra* [13]. As far as we know, compound **2** was first isolated by Yahara et al. [14] from Coptis Rhizoma (the rhizome of *Coptis chinensis*) and as hydrolysis products of phenylethanoid derivatives from *Prunus grayana* [15]. Antioxidant activity of **2**, isolated from *Picrorhiza scrophulariiflora* by Wang et al. [16], showed high scavenging action on hydroxyl radicals (IC_50_ 55.9 μM) produced by H_2_O_2_/Fe^2+^ and on superoxide anion radicals (IC_50_ 86.5 μM) generated by xathine/xanthine oxidase systems.

The chromatographic processes carried out on the methanolic extract of dried, seedless fruits afforded a pure compound known as katsumadin (**3**), which was isolated as a light brown powder and displayed a negative optical rotation value [α]^22^_D_: −4.8 (c 0.1, MeOH). Its molecular formula C_15_O_6_H_14_ was estimated by the molecular ion [M−H]^−^ of *m*/*z* 289.07422 (*m*/*z* calculated of 289.071215) obtained from a high-resolution ESI-QTOF-MS spectrometer (Figure S6), whose carbon number was corroborated by the decoupled ^13^C NMR spectrum (Table S1 and Figure S7). Its structure is composed of two benzene rings: one tetra and the other trisubstituted.

The ^1^H NMR spectrum of **3** revealed a pattern of signals corresponding to an ABX system for the aromatic protons H-6′ (δ 6.73, dd, 8.1, 2.0 Hz), H-5′ (δ 6.77, d, 8.1 Hz), and H-2′ (δ 6.85, d, 2.0 Hz). Furthermore, the presence of a second aromatic ring is evidenced by the presence of two doublet signals at δ 5.88 (H-4″, d, 2.3 Hz) and δ 5.95 (H-6″, d, 2.3 Hz), which clearly indicate a meta-coupled spin pattern belonging to a tetrasubstituted benzene ring (Figure S7). All ^1^H and ^13^C NMR data, including HH COSY, HSQC, and HMBC experiments (Figures S8–S100), are consistent with the katsumadin structure, a biphenylpropanoid first isolated from the seeds of *Alpinia katsumadai*, a plant used in China as an antiemetic to treat stomach disorders [17].

### 2.2. Antiradical ABTS^•+^ Assay

According to the IC_50_ values obtained in the ABTS^•+^ antiradical test, compounds **1** and **2** showed a scavenging capacity higher than **3** and comparable to that of quercetin, according to Tukey’s post hoc test (*p* ˂ 0.05), as shown in Table 1.

### 2.3. In Vivo Antioxidant Test with Saccharomyces cerevisiae and H_2_O_2_

**Figure 2:**
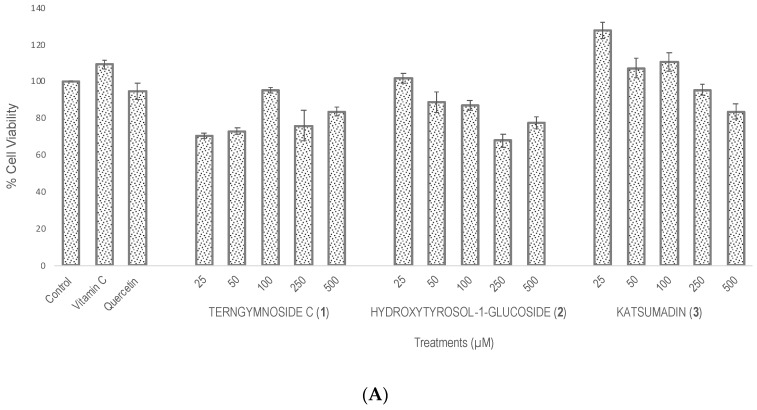

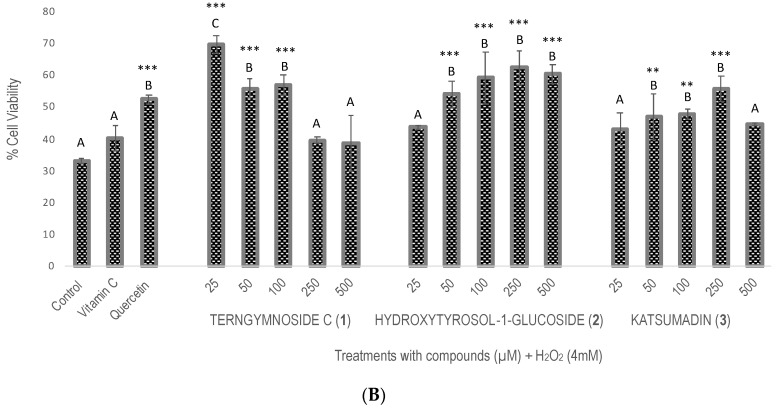
Evaluation of antioxidant activity in *Saccharomyces cerevisiae* growing under H_2_O_2_ oxidative stress. (**A**) Cell viability % after treatments with vitamin C, quercetin, and the different concentrations of **1**, **2**, and **3** (25, 50, 100, 250, and 500 μM). The control did not receive any treatment and was considered to have 100% cell viability. (**B**) Cell viability % of all treatments after exposure with H_2_O_2_ (4 mM). Similar letters do not show significant statistical differences. Treatments with significant statistical differences from the control were represented as ** *p* < 0.01 and *** *p* < 0.001, according to Tukey post hoc test.

Compounds **1**, **2**, and **3** showed different percentages of protection for *Saccharomyces cerevisiae* cell viability after induction of oxidative stress with H_2_O_2_ (4 mM), as shown in Figure 2. In summary, **1** exerted the highest antioxidant protection at 25 μM, maintaining 69.7% cell viability, a value that was significantly higher than the 52% viability observed with quercetin tested at 100 μM as well as with compounds **2** and **3** tested at 25, 50, 100, 250, and 500 μM. The negative control did not receive any type of treatment.

### 2.4. In Silico Antioxidant Analysis to Yeast Aquaporin-3 and Human Aquaporin-7

**Figure 3:**
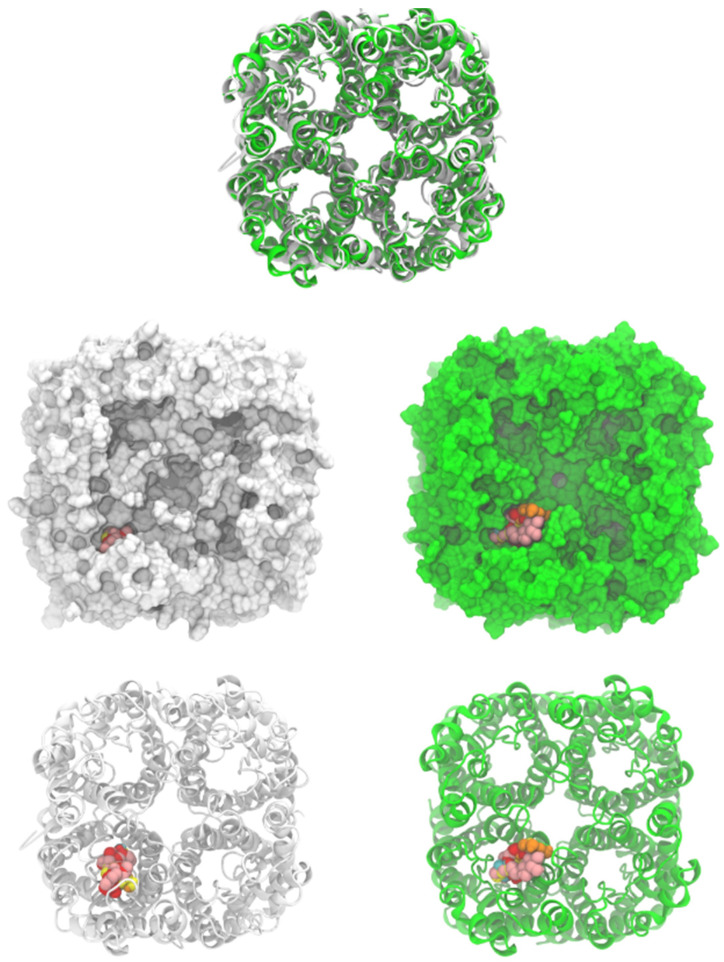
Human aquaporin 7 (white) and *S. cerevisiae* aquaporin 3 model (green, modeled by SwissModel). Cartoons and surface representations of both aquaporins are presented. Docked ligands are shown by spheres.

**Figure 4:**
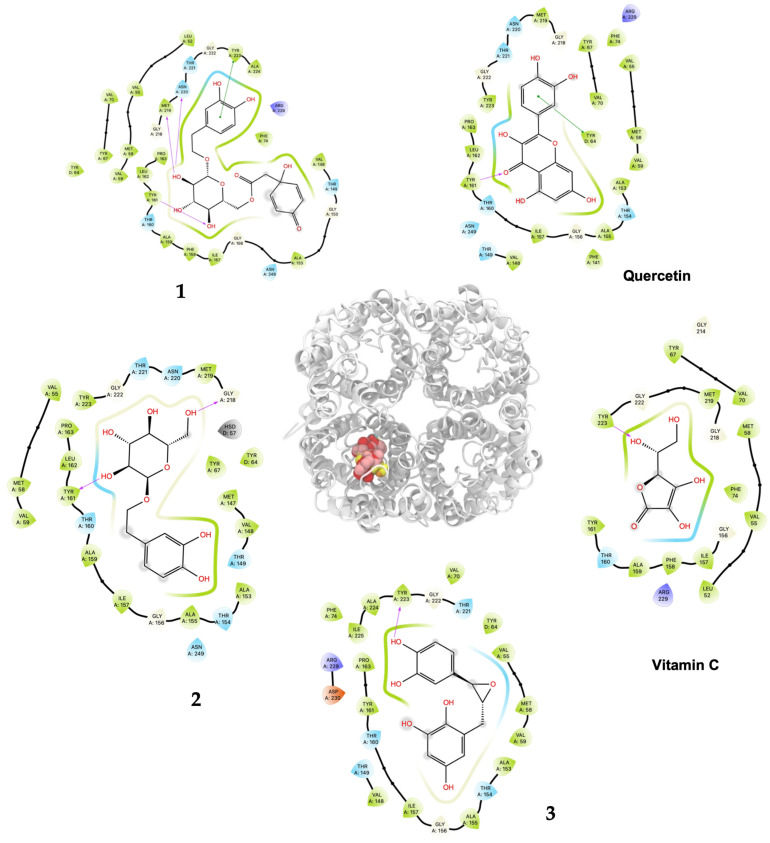
Predicted bonded interactions (red dashed lines) between functional groups of **1**, **2**, and **3**; vitamin C; and quercetin along with the most important amino acid residues of the human AQP7.

**Figure 5:**
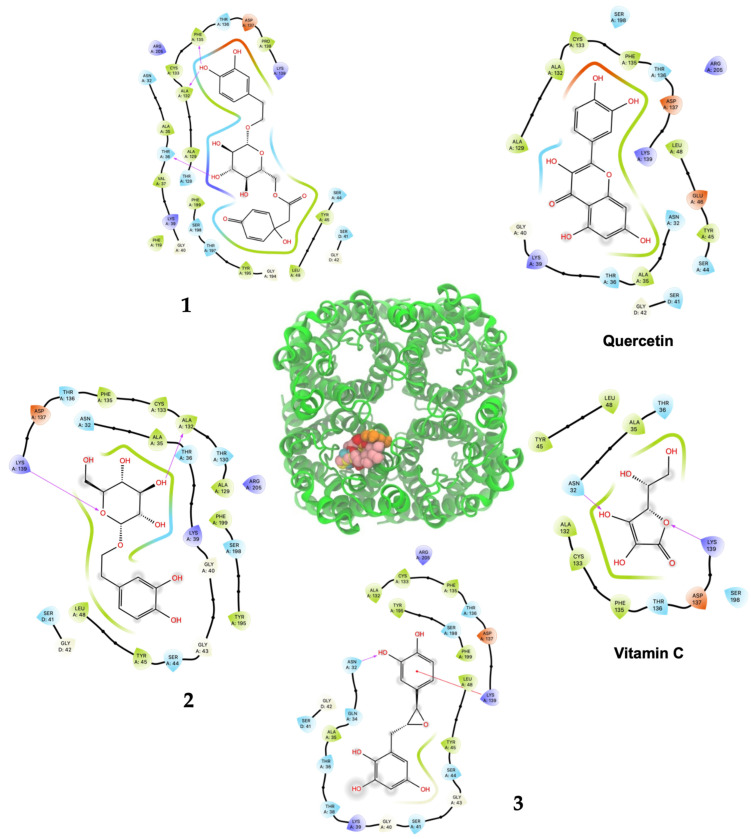
Predicted bonded interactions (red dashed lines) between functional groups of **1**, **2**, and **3**; vitamin C; and quercetin along with the most important amino acid residues of the modeled yeast AQP3 active site region.

As shown in Table 2 and Figure 3, ligand **1** had the better affinity with aquaporin for both the human AQP7 (−9.25 ± 0.11 Kcal/mol) and the modeled yeast AQP3 (−7.39 ± 0.12 Kcal/mol). These results may be explained by the number of hydroxyl groups that increase the number of hydrogen bonds that stabilize the interaction with aquaporin and the number of degrees of freedom that allow **1** to change its conformation, increasing its contacts with aquaporin residues. Also, **2** and **3** displayed strong docking affinity energies of −7.91 ± 0.02 Kcal/mol and −8.03 ± 0.04 Kcal/mol for the human AQP7, respectively. The ligand **2** and **3**, interactions with the yeast AQP3 were lower, with energies values of −6.46 ± 0.05 Kcal/mol and −6.69 ± 0.15 Kcal/mol, respectively. The molecules’ key target complexes with amino acid interactions of the human AQP7 and the modeled yeast AQP3 can be visualized in Figure 4 and Figure 5 and in Table S2.

### 2.5. Cytotoxicity Assay

A cytotoxic study of **1**, **2**, and **3**, using a panel of three cancer cell lines and a normal fibroblast cell line showed that the isolated compounds were not cytotoxic at the highest tested concentration of 20 μg/mL (IC_50_ > 20 μg/mL) in comparison with the positive control camptothecin against HF-6 (IC_50_ 0.030 μg/mL), PC-3 (IC_50_ 0.040 μg/mL), MCF-7 (IC_50_ 0.041 μg/mL), and HFS-30 (IC_50_ 0.60 μg/mL) cell lines, indicating that none of the compounds was considered toxic (IC_50_ values > 4 µg/mL) according to the U.S. National Cancer Institute [18,19].

## 3. Discussion

Traditional herbal medicine plays an important role in maintaining human health, mainly due to its high accessibility worldwide. At the same time, its effectiveness, demonstrated by numerous pharmacological studies, makes it an important support for thousands of scientific investigations [20]. In this sense, even though the antioxidant properties are not described in traditional medicine, the use of plants for diseases related to oxidative stress, as in the case of *T. lineata* used to treat rheumatoid arthritis, has led us to the isolation of interesting antioxidant compounds. The identification for the first time of terngymnoside C (**1**), hydroxytyrosol-1-*O*-glucoside (**2**), and katsumadin (**3**) in *T. lineata*, showing high antioxidant capacity, contributes to a better understanding of the pharmacological properties of this plant used popularly to treat diseases related to oxidative stress. For example, **1** was isolated for the first time from the aerial parts of *T. gymnanthera* and has been documented with analgesic action higher than indomethacin and aspirin, according to Li et al. [9]. This information may contribute to the understanding of the healing action of the infusion of *T. lineata* flowers on rheumatic pain in Mexican traditional medicine. On the other hand, compounds **2** and **3** have been identified in plants such as the olive tree, *Olea europea* L. [21], and *Alpinia katsumadai* [17], respectively.

Countless studies of the benefits of antioxidants for human health are well known; however, there is still little evidence that demonstrates their positive role in patients with rheumatoid arthritis since it is a field that needs further investigation. Nevertheless, there is strong evidence for the role of antioxidants in fighting inflammation, which is intimately linked to the arthritis process [22].

Regarding the properties of **2** and its aglycone, the antioxidant hydroxytyrosol is present in up to 50% of the total phenolic compounds in olive oil [23]. In this context, it was demonstrated that the isomer hydroxytyrosol-4-glucoside is the main phenolic compound in the aqueous extract of the alpeorujo, the byproduct of the process of production of olive oil, followed by hydroxytyrosol and hydroxytyrosol-1-glucoside (**2**), providing to the olive plant multiple benefits attributed to the hydroxytyrosol derivatives [24]. The hydroxytyrosol-4-β-D-*O*-glucoside isomer is an important compound found in olives and derived products [25], and both the aglycone and the glucoside are potent antioxidants, according to their scavenging effect on DPPH radical, with IC_50_ of 0.57 µg/mL and 2.10 µg/mL, respectively, and with the hydroxytyrosol being 3.7 times more active [26].

Our research group has demonstrated the antioxidant activity of the methanolic extracts prepared from different plant organs of *T. lineata* against oxidative stress using the in vitro free radical 2,2′-azino-bis(3-ethylbenzothiazoline-6-sulphonic acid) (ABTS^•+^) scavenger test as well as the H_2_O_2_ protection model with *Saccharomyces cerevisiae*, where the glycosylated phenylethanoid ternstroside B was isolated and tested [27].

In the present work, two known phenylethanoids were isolated (**1** and **2**). Compound **1** (IC_50_ 10.26 µg/mL; 22.00 μM) showed ABTS^•+^ scavenging capacity of the same magnitude as quercetin (IC_50_ 10.25 µg/mL; 33.91 μM). Compound **2** also displayed interesting results with IC_50_ 15.07 µg/mL; 47.64 μM. The biphenylpropanoid **3** proved to be active in the ABTS^•+^ assay with IC_50_ 21.46 µg/mL; 73.93 μM, but it was less potent than **1** and **2**. Furthermore, isolated compounds **1**, **2**, and **3** showed higher antiradical activity than the values we previously reported for the methanolic extracts of leaves (IC_50_ of 33.91 µg/mL) and fruits (IC_50_ of 38.09 µg/mL) from *T. lineata* [27].

The present research on the antiradical capacity and in vivo antioxidant activity shows possible mechanisms of action related to the isolated phenolic natural products, as they can be involved in the regulation and/or maintenance of oxidant homeostasis and the preservation of cell viability. The affinity results of the in silico assay provided for the first time interesting data on a possible mechanism of the direct antioxidant action of **1**, **2**, and **3** with the pore of the model monomers of *S. cerevisiae* AQP3 and human AQP7, which may be involved in the retardation of H_2_O_2_ transport from the extracellular medium. It was demonstrated that the polyphenol rottlerin blocked the permeability of glycerol in AQP3 in a yeast model encoding a human orthodox aquaglyceroporin [28] and also that the action of quercetin prevented and restored the water permeability generated by an oxidative stress (induced by heat) in HeLa cells expressing AQP1, 3, 8, and 11 [29].

Quercetin is a natural product widely documented for its antioxidant properties, for which it has been possible to demonstrate a mechanism of action related to the increase in the expression of aquaporin AQP5 [30] and normalization of the expression of AQP4 [31], which indicates their participation in the modulation of H_2_O_2_ concentrations through these peroxyporins. In general, it has been shown that polyphenols modulate the expression of AQP [32], but until now, there have been few works showing the direct interaction of natural and/or synthetic products on these channel proteins [33,34,35,36].

The mechanism of antioxidant action proposed in the present work comes from the observation of the affinity forces of **1**, **2**, **3**; quercetin; and vitamin C on the pores of AQP3 from *S. cerevisiae* and the human AQP7 by in silico analysis. These values were corroborated by the percentages of viability obtained through the in vivo H_2_O_2_–*Saccharomyces cerevisiae* assay, where the isolated compounds and controls were analyzed at different concentrations, followed by an induction of oxidative stress with H_2_O_2_ (4 mM).

Compound **1** displayed the highest H_2_O_2_–*S. cerevisiae* antioxidant capacity at 25 μM (69.6% cell viability), a value that was higher than quercetin (52.4%) and vitamin C (40.1%), both at 100 μM. These results agreed to the highest in silico affinity for the pores that compose the human AQP7 (−9.25 Kcal/mol) and a similar value to the modeled yeast AQP3 (−7.39 Kcal/mol). On the other hand, **2** and **3** showed in vivo H_2_O_2_–*S. cerevisiae* antioxidant action comparable to quercetin at doses of 50–500 μM and 50–250 μM, respectively, and displayed very similar affinity energies among them for the human AQP7. The vitamin C affinity with the yeast AQP3 and human AQP7 showed the lowest values at −5.30 Kcal/mol and −7.55 Kcal/mol, respectively, which can be related to the experimental results where vitamin C, at a concentration of 100 μM, did not show antioxidant activity in vivo. In this way, the antioxidant protection widely described for vitamin C should not necessarily be related to the direct interaction on the AQP affinity. Nevertheless, it is necessary to carry out more studies on the interaction of these compounds with aquaporins to rule out the possibility of their direct action since there are several isoforms (called AQP0 to AQP12) known in mammals that are located at cellular and subcellular levels and have different degrees of permeability. It is important to emphasize that compounds **1**, **2**, and **3** showed greater in silico affinity for the human AQP7 channel protein pore than for the pore of the AQP3 model of *S. cerevisiae*, which may translate into high antioxidant activity observable in human cells. Furthermore, other cellular mechanisms also contribute to the protective effect against H_2_O_2_-induced oxidative stress. In this sense, it is proposed that compounds **1**, **2**, and **3**, in addition to delaying the passage of H_2_O_2_ through aquaporins, could simultaneously favor the development of a hormetic mechanism at certain doses of 25–100 μM (**1**), 50–500 μM (**2**), and 50–250 μM (**3**), which would be able to activate adaptive cellular responses to stress linked to endogenous signaling pathways that may include kinases, transcription factors that express genes encoding antioxidant enzymes, protein chaperones, phase 2 response (electrophile counterattack response), neurotrophic factors, and other cytoprotective proteins [37]. As an example of this, ferulic acid can regulate the Nrf2/heme oxygenase-1 system, and sulforaphane, curcumin, and resveratrol can activate the Keap1/Nrf2/ARE pathway, which is linked to neuroprotection and cell survival by activation of antioxidant enzymes, respectively [38,39]. Likewise, compounds like hydroxytyrosol, salidroside, tyrosol, and oleuropein could decrease intracellular peroxides, activate caspase-3 activity, and elevate intracellular GSH activity [40]. Terngymnoside C (**1**) was found to have a better affinity for AQP7 (−9.25 Kcal/mol) than any other ligand herein tested (Table 2). Currently, our proposed mechanism is little referenced in natural products, but it may be one of the most significant ways to prevent cell death induced by oxidizing agents from the extracellular environment, as described by Montiel et al. [41]. In a future experimental study, with an adequate amount of the compounds, it will be interesting to evaluate the blocking of aquaporin permeability exerted by **1**, **2**, and **3**. These results appeared to be due to the pi–pi interaction with TYR223 and hydrogen bonds of ligand **1** with TYR161, MET219 and ASN220 of AQP7 (Figure 4 and Table S2). On the other hand, **1** and quercetin had almost the same binding affinity for AQP3 of −7.39 Kcal/mol and −7.58 Kcal/mol, which could explain the high activity of these compounds in the H_2_O_2_–*S. cerevisiae* test. The number of predicted bonded interactions between the modeled AQP3 and the hydroxyl groups of ligand **1** with PHE135, ALA132, and THR36 was lower in contrast to AQP7. Yeast AQP3 is a model based on human AQP7 that could reflect the affinity energies herein obtained. A molecular dynamics simulation of AQP3 is underway, and the results will be published in the near future.

*T. lineata* is a plant that synthesizes interesting antioxidant active metabolites that may counteract oxidative stress through interaction with aquaporins, delaying the entry of H_2_O_2_, which would contribute to maintaining cell viability in the face of oxidative stress. However, it is necessary to perform biochemical permeability tests with potential modulators such as **1**, **2**, and **3** for a clear understanding of this mechanism of action.

Cytotoxic evaluation of **1**, **2**, and **3** in cancer and normal human cell lines provides valuable information that may point to the low toxicity of these phenolic compounds. However, this needs to be explored in the field of toxicology using other in vitro models as well as in vivo assays to validate our findings.

## 4. Materials and Methods

### 4.1. Plant Material

Collection of flower buds and dry seedless fruits of *T. lineata* was carried out in the months of January and February 2013 at the municipality of Huitzilac, Morelos, Mexico (latitude: 19°1′42″ N, longitude: 99°13′88″ W). Specimens collected with voucher number 26349 were identified by Juan Carlos Juárez and deposited at the HUMO Herbarium, CEAMISH, UAEM.

### 4.2. Plant Extracts

Methanolic extracts were prepared from flower buds and dry seedless fruits separately using 200 g of each plant material. For both cases, plant material was previously dried and milled until obtaining a fine powder and subsequently placed in contact with methanol (MeOH) in a 1:5 ratio (g/mL solvent) and sonicated for 20 min (in triplicate). Finally, to obtain the crude extracts of bud petals (43.5 g) and dry seedless fruits (35.5 g), the MeOH extract was filtered (Whatman No. 2 paper) and evaporated under vacuum (Rotavapor*^®^* R-124, Büchi, Flawil, Switzerland) at 40 °C until dryness, affording a yield of 43.5 g and 35.5 g, respectively.

### 4.3. Chromatographic Fractionation and Purification

For the primary chromatographic fractionation of the extracts, a conventional open column method was used. The stationary phase was composed of silica gel 40–60 μm, and the extracts were applied at a proportion of 1:10 (*w*/*w*), and a mobile phase of increasing polarity of CHCl_3_-MeOH was used. The mobile phase ratios were 8:1 to 0:1 for the methanolic extract separation for flower buds, and the ratios were 6:1 to 0:1 for the fractionation of the seedless fruit extract. For the primary fractionation of the methanolic extract of flower buds, a glass column with 50 × 10 cm dimensions was used. A total of 130 fractions with a volume of 15 mL each were collected. Twelve fractions presenting a similar chromatographic profile in thin-layer chromatography were reunited, from which fractions 7 (549 mg) and 10 (452 mg) were selected for the isolation of compounds **1** and **2**, respectively. On the other hand, for the fractionation of the crude extract of dried seedless fruits, a glass column with 120 × 5 cm dimensions was employed. A total of 110 fractions with a volume of 20 mL each were collected. Nine fractions were pooled, from which fraction 8 (46.5 mg) was selected for presenting the major compound **3**. The selected fractions were re-chromatographed with the same mobile phases used in the primary fractionation to obtain fractions with higher purity. After this procedure, all the selected fractions were analyzed by HPLC. The final purifications were achieved through the HPLC core-cutting technique, using a preparative C-18 column (7 μm, 19 × 300 mm) with isocratic phase acetonitrile–H_2_O (HOAc 1%) (80:20 *v*/*v*) at a flow rate of 9 mL/min, affording pure compounds **1** (11 mg), **2** (34 mg), and **3** (19 mg). The purity of these compounds was observed by analytical HPLC and ^1^H and ^13^C NMR spectra. The solvents used for the open column chromatographic process were of analytical and HPLC grade, from J.T. Baker (Philadelphia, PA, USA). TLC analysis was carried out using precoated Si gel 60 F254 aluminum sheets. Silica gel (40−60 μm) was used for open column chromatography. Symmetry C18 columns were used for HPLC analysis on analytical (Waters; 5 μm, 4.6 × 250 mm) and preparative (Waters; 7 μm, 19 × 300 mm) (Massachusetts, USA) scales using HPLC-grade solvents.

### 4.4. Structural Characterization of the Isolated Compounds

Structural identification of **1**, **2**, and **3** was carried out through 1D nuclear magnetic resonance analysis (^1^H and ^13^C) and its 2D modalities, HHCOSY, DEPT, HSQC, and HMBC. A Varian Mercury Plus 400 MHz (9.4 T) spectrometer (Rancho Cordova, CA, USA) was used as well as a Bruker AVANCE III HD 500 MHz (11.74 T) equipment (Billerica, MA, USA). On the other hand, high-resolution mass values afforded the molecular formula of compound **1** through the UHPLC-ESI-QTOF-MS analysis employing an Agilent Ultra-Performance Liquid Chromatography system (1290 Infinity II) equipped with an Agilent Eclipse Plus C18 RRHD column (2.1 × 50 mm, particle size 1.8 μm) (Agilent Technologies, Santa Clara, CA, USA). The column was eluted using deionized water with 0.025% formic acid and acetonitrile, UHPLC-graded, at 85:15 *v*/*v*, with a flow rate of 0.25 mL /min. The mass spectrometry analysis was performed on the Agilent 6545 QTOF apparatus equipped with an electrospray ionization source in positive mode. Compounds 2 and 3 were analyzed by an ACQUITY UPLC H-Class system (Waters-Micromass, Manchester, UK) coupled with a SYNAPT G2-Si Q-TOF hybrid quadrupole time-of-flight instrument (Waters-Micromass, Manchester, UK) equipped with an electrospray (ESI) ionization source (Z-spray) and an additional sprayer for the reference compound (Lock Spray). The chromatographic separation was carried out using a 0.4 mL min^−1^ mobile phase gradient containing 0.01% formic acid in water and methanol programmed from 90:10 to (t = 0 min) 0:100 (t = 8 min). The samples (1 μL of injection volume) were loaded on a KINETEX C18 (1.3 μm, 50 × 2.1 mm) column (Phenomenex, Torrance, CA, USA) heated at 50 °C. The eluates were analyzed by ESI source of the Q-TOF instrument. ESI-HRMS data were recorded in the negative- and positive-ion modes. The source and desolvation temperatures were 120 and 450 °C, respectively. Nitrogen was used as a drying and nebulizing gas at flow rates of 50 and 900 L/h, respectively. Lock mass corrections using [M+H]^+^ and [M−H]^−^ ions at *m*/*z* 556.2771 and 554.2615 of a leucine–enkephalin solution (50 pg μL^−1^ in 50:50 acetonitrile/water + 0.1% formic acid) were used for accurate mass determinations. Data acquisition and processing were performed with MassLynx 4.1 software [42].

### 4.5. Antiradical ABTS^•+^ Assay

The ABTS assay was adapted from Re et al. [43]. The assay was performed in 96-well microplates, where 20 μL of the compounds was added to 230 μL of the ethanolic ABTS^•+^ solution previously adjusted to an absorbance of 0.70 (±0.1) at 754 nm. Different concentrations of the compounds at 156.2, 312.5, 625, 1250, and 2500 μg/mL were prepared in methanol. The absorbance value was read at 30 °C for a period of 1, 4, and 6 min after mixing. For the negative and positive controls, 20 μL of methanol (vehicle) and 20 μL of quercetin (positive control) were added to 230 μL of ABTS^•+^ solution. Finally, all tested results were expressed as median inhibitory concentrations (IC_50_).

### 4.6. In Vivo H_2_O_2_–Antioxidant Assay Using Saccharomyces cerevisiae

This assay was adapted from the work of Golla and Bhimathati [44]. A wild-type *Saccharomyces cerevisiae* strain, which was donated by Dr. Omar Ayala Homero Pantoja from the Institute of Biotechnology at the National Autonomous University of Mexico (UNAM), Mexico, was grown in liquid yeast peptone dextrose (YPD) medium at 28 °C and 200 rpm. Cells were collected at the exponential growth phase, where value 1 to the optical density at 600 nm of 1 mL medium corresponded to 1 × 10^8^ cells/mL, which were placed in a 24-well plates with compounds **1**, **2**, and **3** at 25, 50, 100, 250, and 500 μM. Cells were then prepared in Milli-Q^®^ water (Merck, Darmstadt, Germany) and incubated for 1 h at 28 °C and 170 rpm. Quercetin and vitamin C, both at 100 μM, were employed as positive controls. Subsequently, the treated cells with compounds and the control were stressed by the addition of 4 mM H_2_O_2_ prepared in a phosphate-buffered saline (PBS) solution at pH 7.4 and incubated again for 2 h at the same previous conditions. Cell viability was determined by counting colony-forming units (CFU). For that purpose, cell dilutions of 1:10,000 were obtained using YPD liquid medium, seeded in YPD solid medium plates, and incubated for 48 h at 28 °C. All experiments were conducted in triplicate and reported as the mean cell percentage of survival, which represents the protective antioxidant activity. Cell viability without any treatment (negative control) corresponded to 100% viability (100 V). Protective antioxidant percentage (% PA) of the samples was calculated considering the cell viability after treatment with compounds (% CVC) during 1 h and then exposed to H_2_O_2_ (% CVP) for 2 h, as expressed in the following formula: (100V/% CVC) × % CVP. Chemicals for bioassays were purchased from Sigma-Aldrich (St. Louis, MO, USA).

### 4.7. In Silico Antioxidant Analysis with Aquaporin-3 and -7

*Saccharomyces cerevisiae* aquaporin-3 was modeled, using human aquaporin 7 (6KXW, chain A), by the SwissModel server [45]. The yeast AQP3 sequence (ID: P43549) was obtained from the Uniprot database [46]. The model had two quality parameters: the global model quality estimate (GMQE) of 0.7 and the QMEANDisCo global score of 0.63 ± 0.05. The parameters indicate the confidence of the model. Residues from 337 to 617 were used to build the model. Human AQP 7 (PDB ID: 6KXW) was used to build the yeast tetrameric aquaporin on the SwissModel server [47]. Human and *S. cerevisiae* structures were uploaded on the CHARMM-GUI server to assign hydrogens and charges to all atoms with CHARMM 36 potential [48,49,50]. Both structures were energy-minimized with 100 steps of the steepest descent method, as implemented on CHARMM 44b1 [51]. Both structures were oriented (Figure 3), and it was noted that there was a high resemblance between both structures even when the sequence percentage conservation was 37.4%.

The *S. cerevisiae* tetramer structure was built using a modeled monomer and located in equivalent monomer positions on the human AQP7 structure. Based on this, we docked the ligands only on one monomer of human and *S. cerevisiae* structures. Quercetin, vitamin C, and **1**, **2**, and **3** were docked on one monomer of human and *S. cerevisiae* aquaporins using the Autodock Vina 1.2.5 [52,53] and the Vina scoring function. All systems were prepared on Chimera UCSF (alpha version 1.18) using the Autodock Vina tool included [54,55]. All ligands were built on MarvinSketch 20.16 (Chemaxon, Budapest, Hungary), and the conformer with lower energy was used as the initial ligand. The grid box center was located at (30.93, 25.48, and 63.86) with a size of (20 × 20 × 50 Å^3^). The grid box size was large enough to cover the monomer pore completely. In total, 100 independent docking experiments were realized on each structure. All outputs were clustered by their root mean squared deviation (RMSD) within a 2 Å value. The average affinity energy and its corresponding standard deviation are reported. One representative structure of the most populated cluster was used to generate the interaction maps. All images were generated with Visual Molecular Dynamics (VMD) [56] and protein/ligand interaction maps were obtained with Maestro from Schrodinger (2023-3) [57].

### 4.8. Cytotoxicity Assay

In vitro cytotoxic activity was determined by the sulforhodamine B protein staining assay using the HF-6 (colon), PC-3 (prostate), and MCF-7 (breast) cancer cell lines as well as toward HFS-30 fibroblast normal skin cells. Cell cultures were maintained in RPMI-1640 medium (Merck, Darmstadt, Germany) supplemented with 10% fetal bovine serum (Biowest, South America origin, Bradenton, FL, USA), 5000 units/mL penicillin, 5 mg/mL streptomycin, 7.5% NaHCO_3_, and cultured in a 96-well microtiter plate at 37 °C in 5% CO_2_ at 100% humidity. Different concentrations of **1**, **2**, and **3** at 0.16, 0.8, 4, and 20 μg/mL, prepared in RPMI medium supplemented (Biowest, Bradenton, FL, USA) with 0.025% DMSO, were assayed with the cells at the log phase of growth. The cell viability was determined by the optical density at 590 nm using an ELISA-Reader (SpectraMax^®^ iD3 Plus 384, Molecular Devices, San Jose, CA, USA). After the incubation period of 72, the IC_50_ values were estimated from a semilog plot of the compound concentrations (μg/mL) against the percentage of viable cells. Compounds with inhibitory concentrations ≤4 μg/mL were considered active according to the National Cancer Institute (NCI) guidelines [18,19]. Camptothecin (Sigma-Aldrich, Darmstadt, Germany) was used as positive control.

### 4.9. Statistical Analysis

All assays were conducted in triplicate. Mean ± SD values were calculated, and the data were subjected to ANOVA followed by Dunnett’s test to compare controls with treatments. Also, the Tukey’s test for multiple comparisons of the means was carried out to find differences at *p* < 0.05 between treatments. The statistical analysis of the results was performed using the SAS 9.5 program.

## 5. Conclusions

Polyphenolic compounds **1**, **2**, and **3** were described for the first time in *T. lineata*, so this work contributes significantly to the phytochemical knowledge of the plant.

*T. lineata* is a plant that synthesizes interesting antioxidant active metabolites that may counteract H_2_O_2_-mediated oxidative stress through their interaction with the pore of the monomers from the aquaporins AQP3 and AQP7, which favors the delay of H_2_O_2_ entry and allows the maintenance of cell viability. However, it is necessary to corroborate this mechanism based on biochemical permeability tests with potential modulators such as **1**, **2**, and **3** for validation. On the other hand, the high antioxidant potential of the isolated compounds as well as their low toxicity opens interesting alternatives that could be effective and safe for the treatment of diseases such as rheumatoid arthritis that are related to the development of oxidative stress.

## Figures and Tables

**Table 1:** Inhibitory activity of the radical ABTS^•+^.

Compounds	Inhibition IC_50_ (µg/mL) *
**1**	10.26 ± 0.43 (22.00 ± 0.9 μM) ^A^
**2**	15.07 ± 0.88 (47.64 ± 2.9 μM) ^A^
**3**	21.46 ± 2.07 (73.93 ± 7.1 μM) ^B^
Quercetin	10.25 ± 1.45 (33.91 ± 4.8 μM) ^A^

* Similar letters do not show significant statistical differences according to Tukey *p* < 0.05.

**Table 2:** The docking affinity energies of the human aquaporin 7 and the modeled *S. cerevisiae* aquaporin 3 model and ligand herein studied are presented (kcal/mol). Average energies and standard deviations are shown.

	Quercetin	Vitamin C	1	2	3
AQP7 human	−8.00 ± 0.01 (100)	−7.55 ± 1.60 (100)	−9.25 ± 0.11 (86)	−7.91 ± 0.02 (100)	−8.03 ± 0.04 (100)
AQP3 *S. cerevisiae*	−7.58 ± 0.01 (100)	−5.30 ± 0.03 (100)	−7.39 ± 0.12 (86)	−6.46 ± 0.05 (75)	−6.69 ± 0.15 (87)

Numbers in parenthesis are the number of members of the most populated cluster.

## Data Availability

The data presented in this study are available on request from the corresponding authors.

## Supplementary Materials

The supporting information can be downloaded at: https://www.mdpi.com/article/10.3390/plants13162223/s1, Figure S1: UHPLC-ESI-QTOF-MS analysis of **1**, showing the high-resolution mass spectra (above) and the UHPLC chromatogram (below). The molecular formula of **1** (C_22_O_11_H_26_) was calculated through the molecular ion [M+H]^+^ 467.1547 and the adduct [M+Na]^+^ 489.1372; Figure S2: ^1^H NMR (A) and ^13^C DEPT 135 (B) NMR spectra of **1** recorded at 400 MHz apparatus in CD_3_OD; Figure S3: HMBC spectra of **1** recorded at 400 MHz apparatus in CD_3_OD; Figure S4: ESI-QTOF-MS analysis of **2**, showing the molecular ion [M−H]^−^ of *m*/*z* 315.10913 (spectrum above) and the adduct [M+Na]^+^ of m/z 339.34766 (spectrum below); Figure S5: ^1^H NMR (A) and ^13^C NMR (B) spectra of **2** recorded at 400 MHz apparatus in CD_3_OD; Figure S6: ESI-QTOF-MS analysis of **3**, showing the molecular ion [M−H]^−^ of *m*/*z* 289.07422; Figure S7: ^1^H NMR (A) and ^13^C NMR (B) spectra of **3** recorded at 500 MHz apparatus in CD_3_OD; Figure S8: HHCOSY spectra of **3** recorded at 400 MHz apparatus in CD_3_OD; Figure S9: HSQC spectra of **3** recorded at 400 MHz apparatus in CD_3_OD; Figure S10: HMBC spectra of **3** recorded at 400 MHz apparatus in CD_3_OD; Table S1: NMR data of terngymnoside C (**1**) *, hydroxytyrosol 1-glucoside (**2**) *, and katsumandin (**3**) ** at * 400 MHz (^1^H) and ** 500 MHz (^13^C) in CD_3_OD; Table S2: Binding sites for each ligand herein tested. Amino acids were aligned previously. Docking studies were conducted on a single monomer. Those residues that belong to a contiguous monomer are shown with a *.
