# Supplementary material for: Antioxidant Active Phytochemicals in Ternstroemia lineata Explained by Aquaporin Mechanisms
Source: Plants (Basel). 2024 Aug 10;13(16):2223. doi: 10.3390/plants13162223 (PMC11360478; doi:10.3390/plants13162223)
Supplement: Supplementary file 1 [file plants-13-02223-s001.zip › plants-3105866-supplementary.pdf]

Antioxidant active phytochemicals in *Ternstroemia lineata* explained by aquaporin mechanisms

Nahim Salgado-Medrano [1,2], César Millán-Pacheco [3], Verónica Rodríguez-López [3], Lucía Corona-Sánchez [3], François Mesnard [4], Roland Molinié [4], Eleazar León-Álvarez[1], María Luisa Villarreal [1,*] and Alexandre Toshirrico Cardoso-Taketa [1,*]

Supplementary Materials

Figures S1-S10

Tables S1 and S2

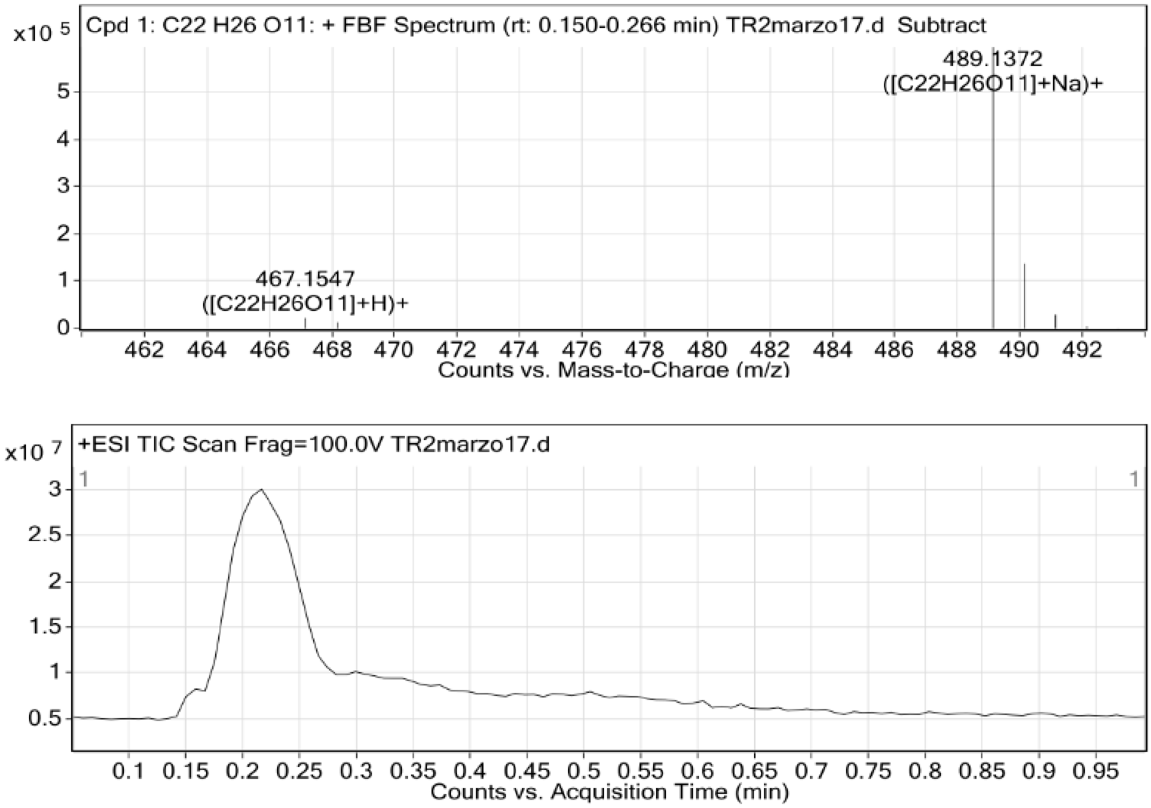

Figure S1. UHPLC-ESI-QTOF-MS analysis of **1**, showing the high-resolution mass spectra (above) and the UHPLC chromatogram (below). The molecular formula of **1** ($C_{22}O_{11}H_{26}$) was calculated through the molecular ion $[M+H]^+$ 467.1547 and the adduct $[M+Na]^+$ 489.1372.

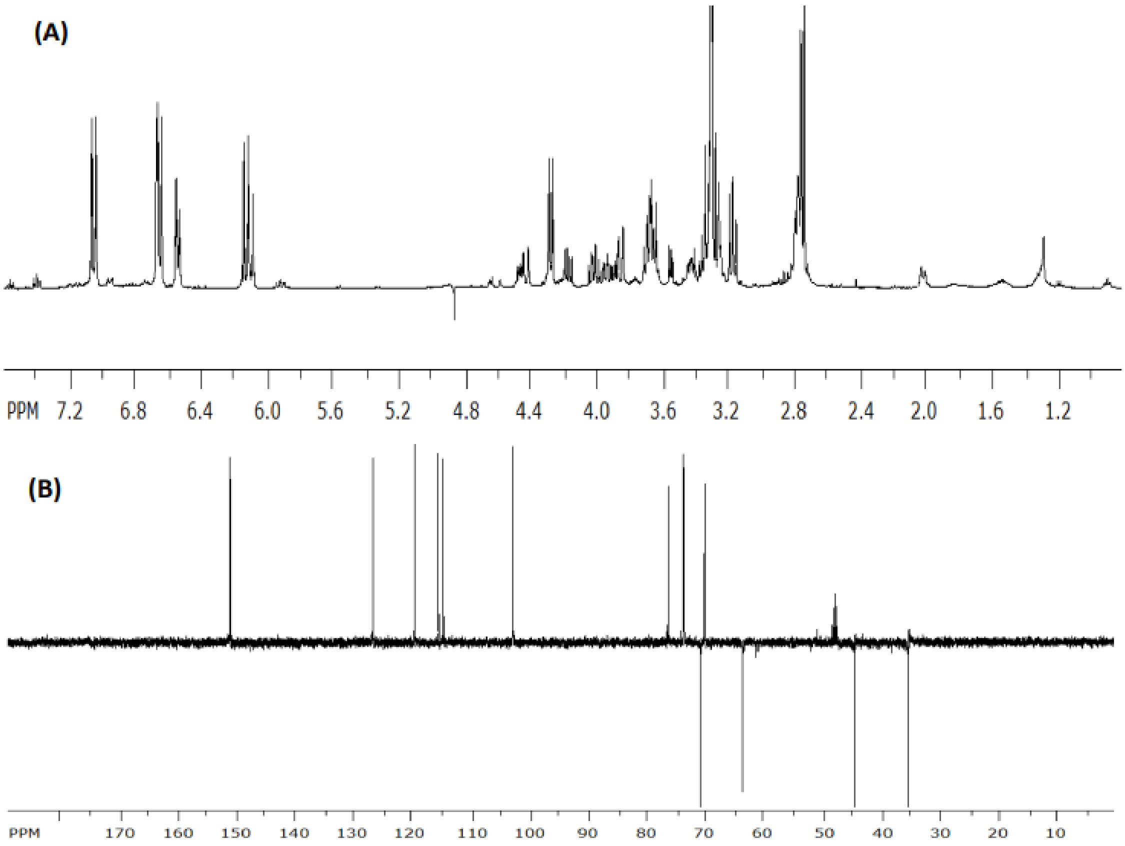

Figure S2. ^{1}H NMR (A) and ^{13}C DEPT 135 (B) NMR spectra of **1** recorded at 400 MHz apparatus in CD_3OD.

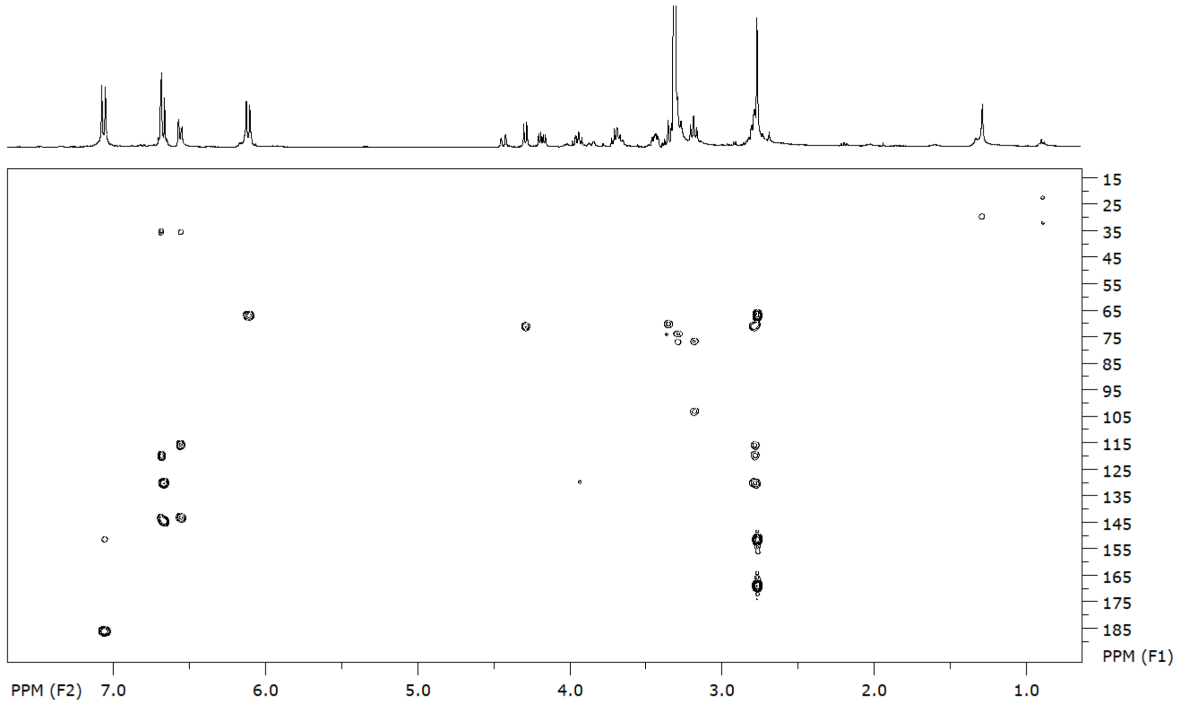

Figure S3. HMBC spectra of **1** recorded at 400 MHz apparatus in CD_3OD.

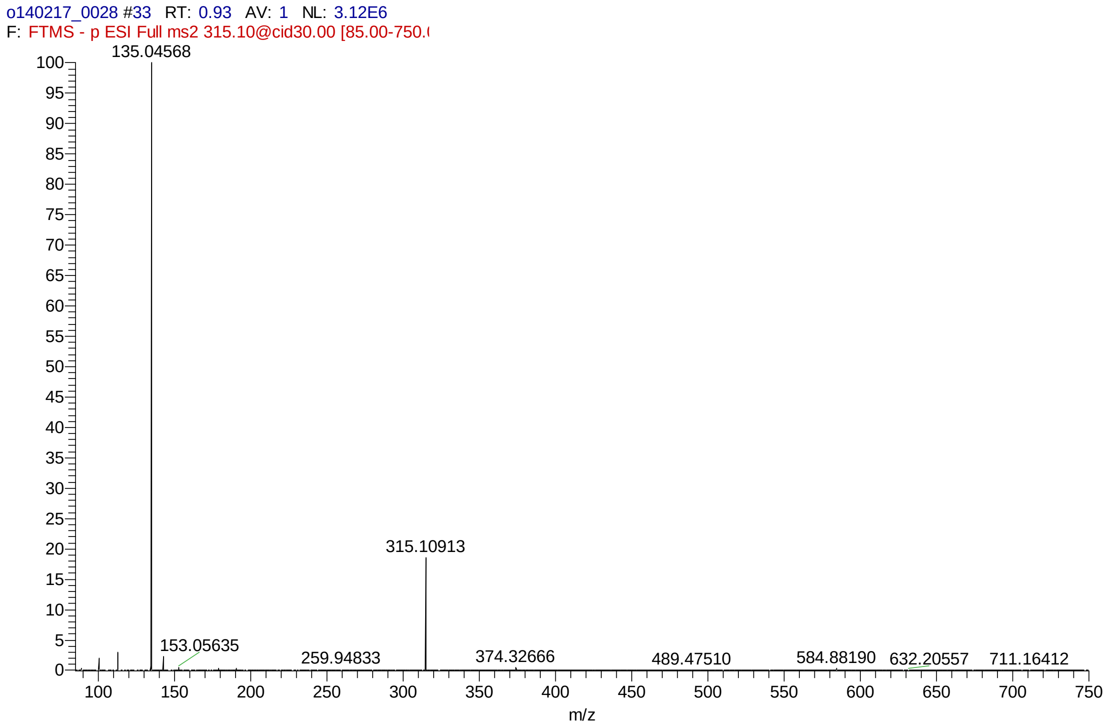

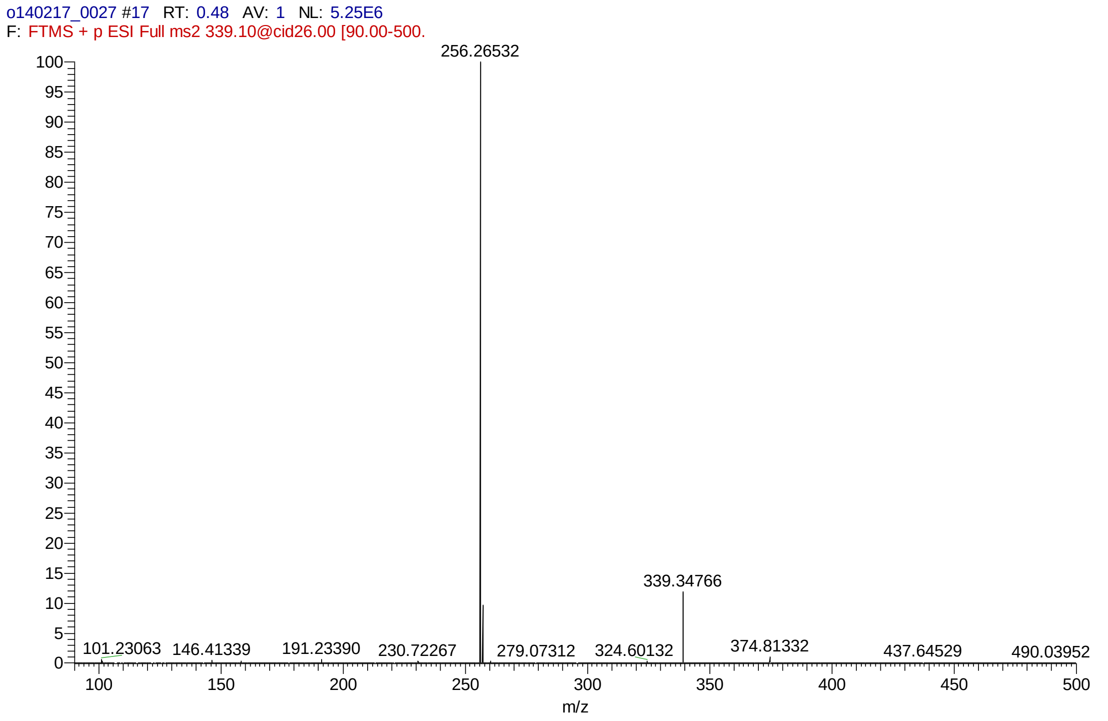

Figure S4. ESI-QTOF-MS analysis of **2**, showing the molecular ion $[M\text{-}H]^{-}$ of m/z 315.10913 (spectrum above) and the adduct $[M+Na]^{+}$ of m/z 339.34766 (spectrum below).

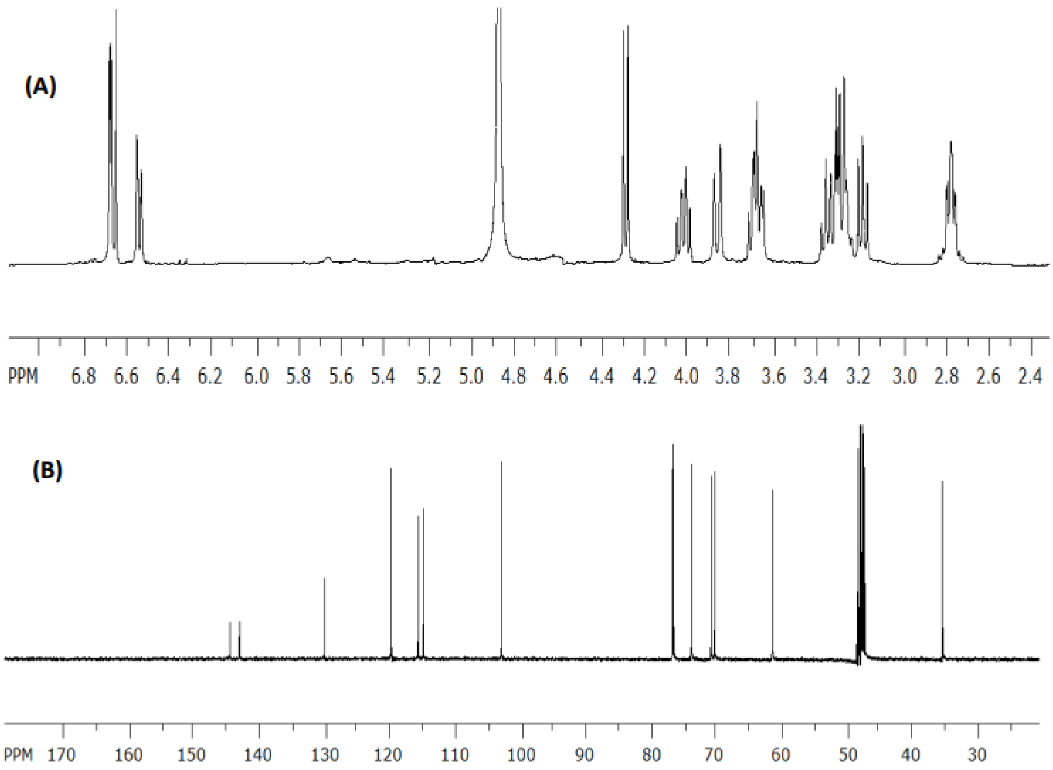

Figure S5. ^{1}H NMR (A) and ^{13}C NMR (B) spectra of **2** recorded at 400 MHz apparatus in CD_3OD.

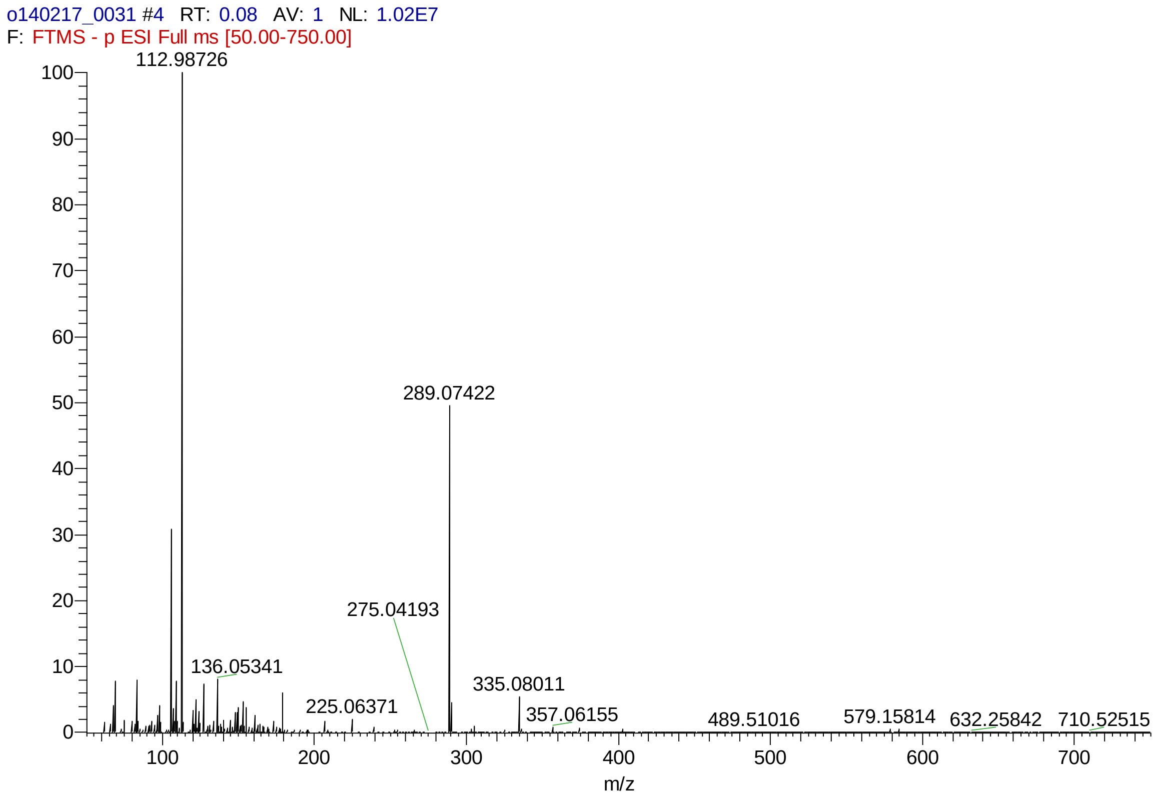

Figure S6. ESI-QTOF-MS analysis of **3**, showing the molecular ion $[M-H]^-$ of m/z 289.07422.

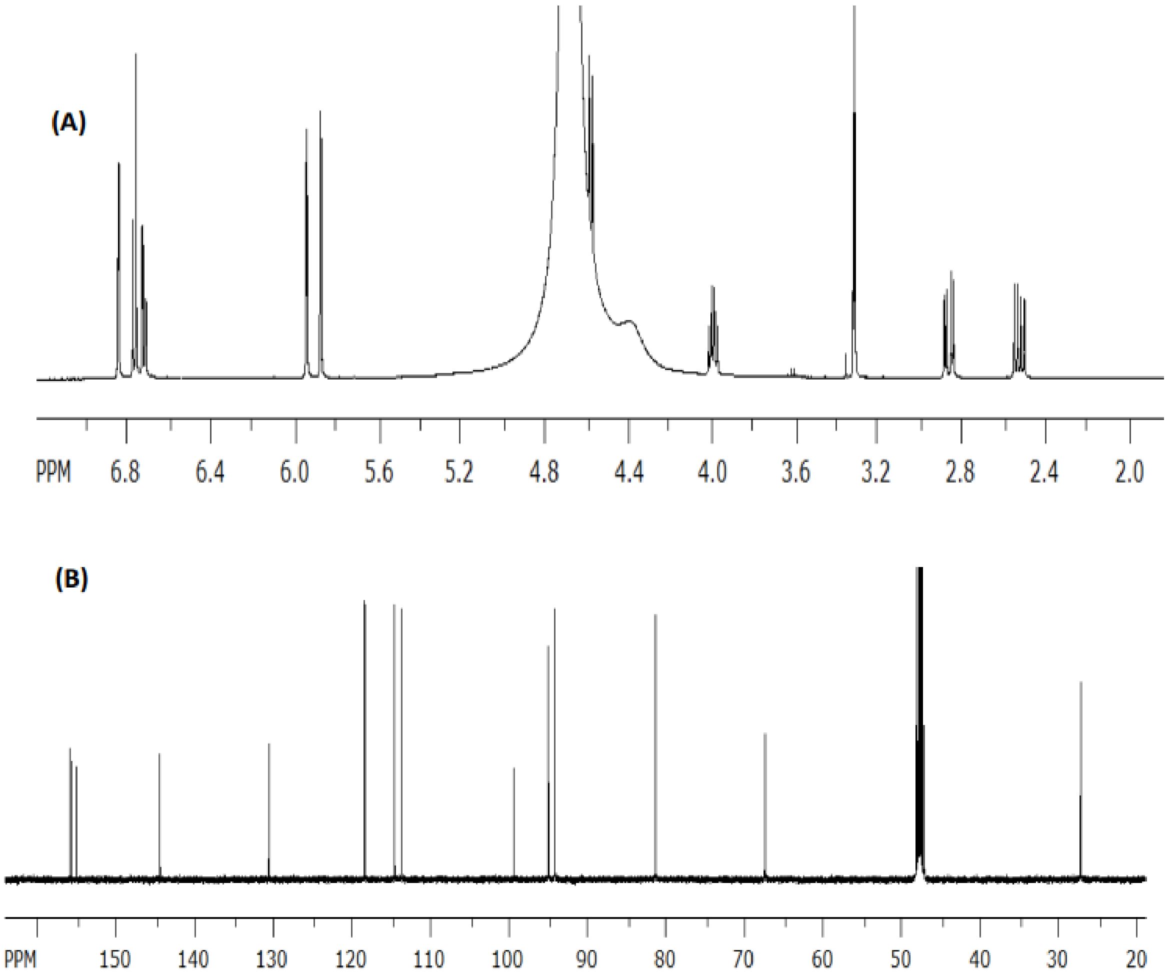

Figure S7. ^{1}H NMR (A) and ^{13}C NMR (B) spectra of **3** recorded at 500 MHz apparatus in CD_3OD.

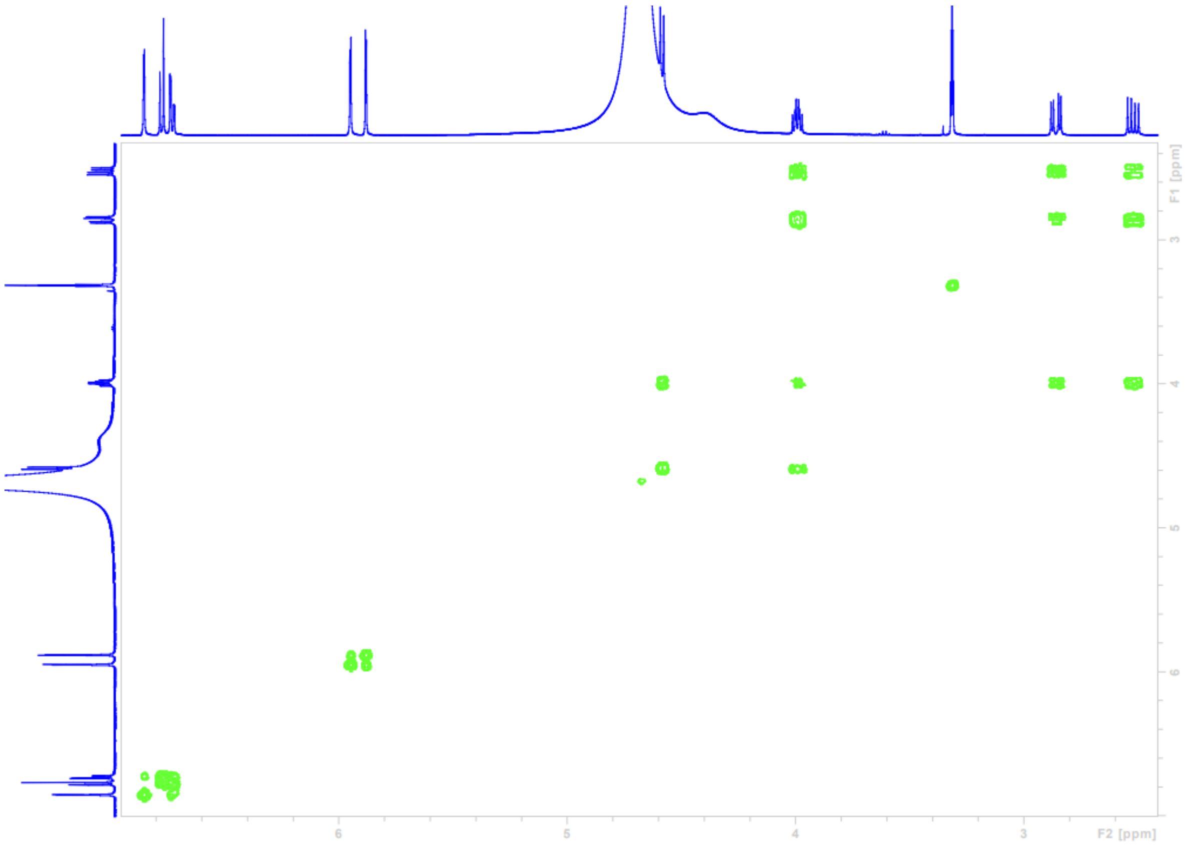

Figure S8. HHCOSY spectra of **3** recorded at 400 MHz apparatus in CD_3OD.

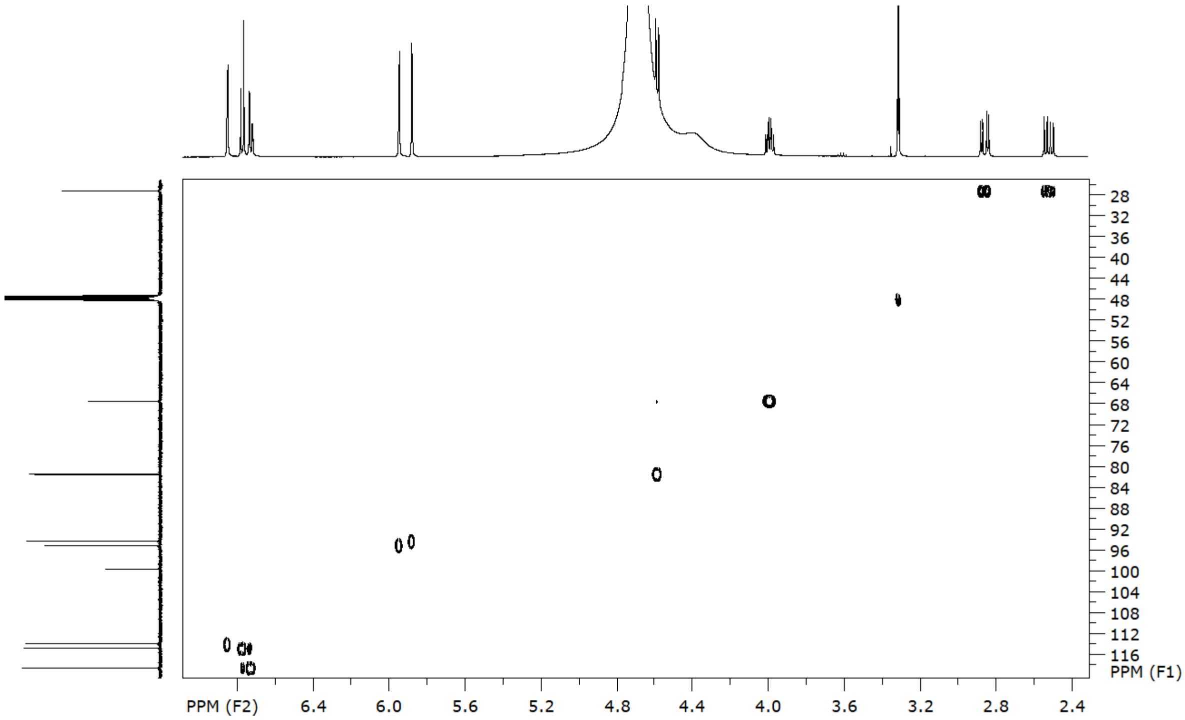

Figure S9. HSQC spectra of **3** recorded at 400 MHz apparatus in CD_3OD.

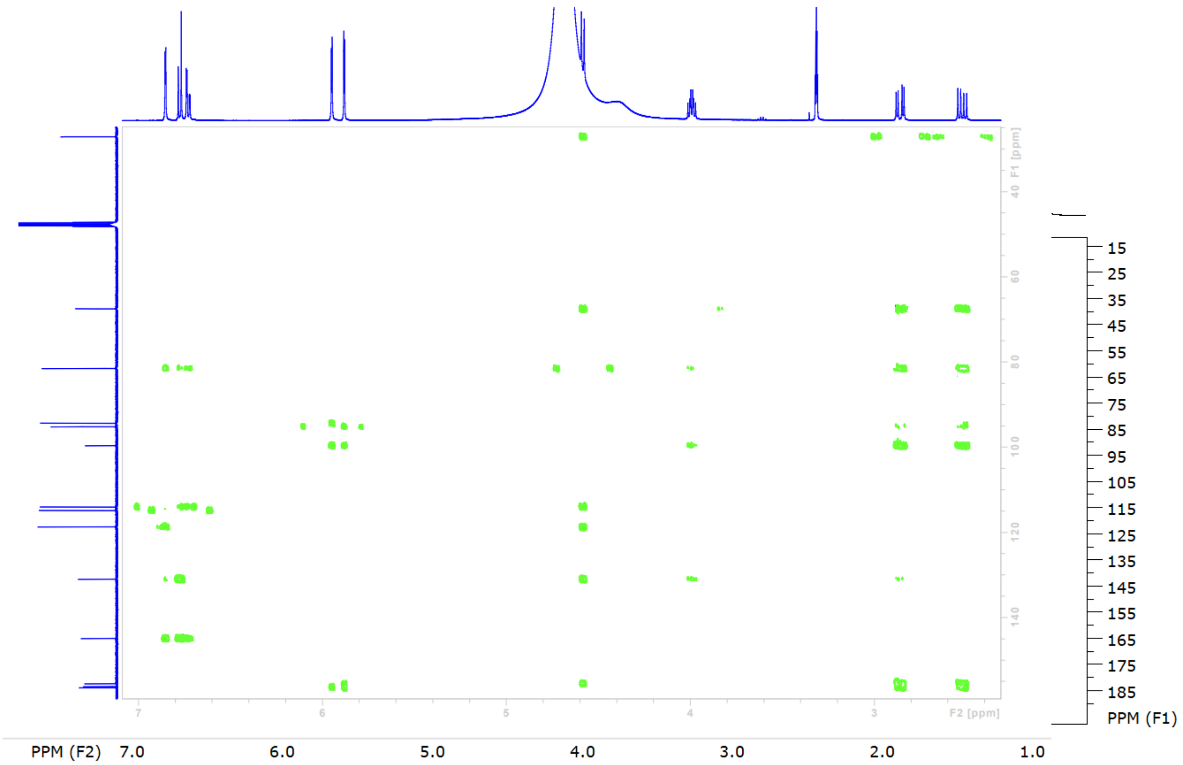

Figure S10. HMBC spectra of **3** recorded at 400 MHz apparatus in CD_3OD.

Table S1. NMR data of terngymnoside C (**1**)*, hydroxytyrosol 1-glucoside (**2**)*, and katsumandin (**3**)** at *400MHz (^{1}H) and **500 MHz (^{13}C) in CD_3OD.

N°	1		2		N°	3	
	δ_H, mult, *J* = Hz	δ_C	δ_H, mult, *J* = Hz	δ_C		δ_H, mult, *J* = Hz	δ_C
1	4.29, d, 8.0	104.54	4.28, d, 8.0	104.41	1	4.58, d, 7.6	83.0
2	3.18, t, 8.0	75.29	3.18, t, 8.0	75.19	2	3.99, m	69.0
3	3.28, ov.	71.81	3.30, ov.	71.68	3	2.52, dd, 16.2, 8.0	28.6
4	3.34, t, 8.8	78.10	3.35, t, 8.4	78.19	1´		132.5
5	3.42, ov.	75.29	3.26, ov.	77.99	2´	6.85, d, 2.0	116.3
6	4.17, dd, 12.0, 6.0<br>4.43, dd, 12.0, 2.0	65.19	3.64, ov.<br>3.86, dd, 12.0, 2.0	62.80	3´		146.3
1´		131.68		131.59	4´		146.3
2´	6.68, d, 2.1	117.21	6.68, d, 2.1	117.21	5´	6.77, d, 8.2	115.5
3´		146.31		146.18	6´	6.73, dd, 8.2, 2.0	120.2
4´		144.80		144.72	1´´		101.1
5´	6.66, d, 8.0	116.45	6.67, d, 8.0	116.41	2´´		157.0
6´	6.54, dd, 8.0, 2.1	121.40	6.55, dd, 8.0, 2.1	121.37	3´´		157.7
7´	2.77, m	36.74	2.80, m	36.64	4´´	5.84, d, 2.3<br>5.88, d, 2.3	95.8
8´	3.68, m<br>4.02, m	72.25	3.66, m<br>4.02, m	72.20	5´´		157.9
1´´		68.24			6´´	5.95, d, 2.3	96.7
2´´	7.06, d, 10.1	152.90					
3´´	6.14, d, 10.1	128.42					
4´´		187.59					
5´´	6.14, d, 10.1	128.42					

6´´	7.06, d, 10.1	152.90
7´´	2.74, m	45.90
8´´		170.49

Table S2. Binding sites for each ligand herein tested. Amino acids were aligned previously. Docking studies were conducted on a single monomer. Those residues that belong to a contiguous monomer are shown with a *.

AQP3		1	2	3	Q	VC	AQP7		1	2	3	Q	VC
							LEU	52	*				*
ASN	32	*	*	*	*	*	VAL	55	*	*	*	*	*
GLN	34			*			HIS*	57		*			
ALA	35	*	*	*	*	*	MET	58	*	*	*	*	*
THR	36	*	*	*	*	*	VAL	59	*	*	*	*	
VAL	37	*											
THR	38			*									
LYS	39	*	*	*	*								
GLY	40	*	*	*	*								
SER*	41	*	*	*	*								
SER	41			*									
GLY*	42	*	*	*	*		TYR*	64	*	*	*	*	
GLY	43		*	*									
SER	44	*	*	*	*								
**TYR**	**45**	*	*	*	*	*	**TYR**	**67**	*	*		*	*
GLU	46				*								
LEU	48	*	*	*	*	*	VAL	70	*		*	*	*
							PHE	74	*		*	*	*
PHE	119	*					PHE	141				*	
							MET	147		*			
							VAL	148	*	*	*	*	
							THR	149	*	*	*	*	
THR	128	*					GLY	150	*				
**ALA**	**129**	*	*		*		**ALA**	**153**	*	*	*	*	
**THR**	**130**		*				**THR**	**154**		*	*	*	
							ALA	155		*	*	*	
							GLY	156	*	*	*	*	*

ALA	132	*	*	*	*	*	ILE	157	*	*	*	*	*
CYS	133	*	*	*	*	*	PHE	158	*				*
							ALA	159	*	*			*
PHE	135	*	*	*	*	*	THR	160	*	*	*	*	*
THR	136	*	*	*	*	*	TYR	161	*	*	*	*	*
ASP	137	*	*	*	*	*	LEU	162	*	*		*	
**PRO**	**138**	*					**PRO**	**163**	*	*	*	*	
LYS	139	*	*	*	*	*							
							GLY	214					*
**GLY**	**194**	*					**GLY**	**218**	*	*		*	*
TYR	195	*	*	*			MET	219	*	*		*	*
							ASN	220	*	*		*	
**THR**	**197**	*					**THR**	**221**	*	*	*	*	
SER	198	*	*	*	*	*	GLY	222	*	*	*	*	*
PHE	199	*	*	*			TYR	223	*	*	*	*	*
							ALA	224	*		*		
							ILE	225			*		
**ARG**	**205**	*	*	*	*		**ARG**	**229**	*		*	*	*
							ASP	230			*		
							ASN	249	*	*		*	

Residue's locations are based on sequence alignment (not shown). * Indicates residues on a chain contiguous to the original docked chain. Residues that are the same on both sequences are in bold.
